# 

**Published:** 1902-07-26

**Authors:** 


### NOTICE TO CORRESPONDENTS.

All MSS., Letters, Books for Review, and other matters intended for the Editor should be addressed THE EDITOR, "THE HOSPITAL," THE HOSPITAL BUILDING, 28 & 29 SOUTHAMPTON STREET, STRAND, W.C.

The Editor cannot undertake to return rejected MS., even when accompanied by stamped directed envelope.

**Notice to Advertisers and Subscribers.**

All Advertisements, Orders for Copies of THE HOSPITAL, and Business Communications should be addressed to **THE MANAGER, (Not to the Editor)**, THE HOSPITAL, 28 & 29 Southampton Street, Strand, London, W.C.

The Cost of Subscription, post free, is as follows.—Per week, 2½d.; per quarter, 2s. 8d.; per half-year, 5s. 3d.; per annum, 10s. 6d. Foreign:—Per annum, 15s. 2d., payable, in all cases, in advance.

**NOTICE.—Vol. XXXI. of "The Hospital" (October 1901, to March 1902), in handsome cloth binding is now on sale at the office of this Journal, price, 7s. 6d. Cases for binding the Numbers contained in this Volume 1s. 6d. Index to Vol. XXXI., 2½d., post free.**

**The Monthly Part for August will be ready on August 1. Price 6d., by post 8d.**

### BOOKS RECEIVED.

W. B. Saunders and Co.

"The American Illustrated medical Dictionary." By W. A. Newman Dorland, A.M., M.D.

The Sanitary Publishing Co.

"Digest of Public Health Cases." By J. E. R. Stephene, Barrister-at law.

Longmans, Green, and Co.

"The Earth in Relation to Contagia." By George Vivian Poore, M.D.Lond., F.R.C.P.

Swan, Sonnenschein and Co.

"Garden Cities of To-morrow." By Ebenezer Howard.

### SCRAPS AND GLEANINGS.

Hospital Sunday at Winterton this year realised £14 odd, which amount has been handed to the Hull Royal Infirmary.

The Hospital Saturday collections in Birmingham this year have far exceeded those of last year. For the second time in the history of the Birmingham Hospital Saturday Fund the street boxes were dispensed with.

The takings of the two days' sports and pageant in connection with Hospital Saturday in Aberdeen this year, amounted to about £200, or about £100 less than the total last year. It is stated to be doubtful whether after paying expenses there will be any balance left.

The new small-pox hospital at Purdysburn is now complete. There is accommodation in the hospital proper for 32 patients—eight patients in each of the four wards. The building is of corrugated iron with foundations of brick and cement, and it was put up in the short space of time between the 6th and 25th June.

### THE STUDENT OF MEDICINE.

### APPOINTMENTS.

**E. Farquhar Buzzard**, M.B.Oxon., M.R.C.P.Lond., appointed Pathologist to the National Hospital for the Paralysed and Epileptic. **John Hy. Chaldecott**, L.R.C.P.Lond., F.F.P.S.Glas., appointed Honorary Anæsthetist to the Italian Hospital, Queen Square, W.C. **T.S. Collin**, L.S.A., appointed Public Vaccinator and Medical Officer for the Wallingfen District of the Howden Union. **J. McCassoll**, M.B., B.Ch., R.U.I., appointed Casualty Medical Officer of the Middlesex Hospital. **James Murray**, M.B., C.M.Aberd., appointed Medical Officer to His Majesty's Prison, Birmingham. **Jones Powell**, M.B.Lond., appointed Medical Officer for the No. 2 District of the Swansea Union. **John Walter Pridmore**, M.R.C.S., L.R.C.P.Lond., appointed District Medical Officer and Public Vaccinator of the Ryde District of the Isle of Wight Union. **F. W. Rigby**, M.B., C.M.Edin., appointed Certifying Factory Surgeon for the Bacup District of Lancashire. **M. McIntyre Sinclair**, M.D.Glasg., D.P.H.Camb., appointed Resident Medical Officer to the New Sanatorium for Phthisical Patients of the Poorer Classes, Blue Mountain, New South Wales. **John Aldren Wright**, M.A. M.D., appointed an Assistant Physician to the Addenbrooke Hospital, Cambridge.

### "The Hospital" Institutional Library.

"Hospitals and Asylums of the World." 4 Vols., with a Portfolio of Plans, complete £12 12s.
"Burdett's Hospitals and Charities, 1902." 5s.
"Cottage Hospitals, General, Fever, and Convalescent." 10s. 6d.
"Hospital Expenditure: The Commissariat." 2s. 6d.
"A Legal Handbook for the Use of Hospital Authorities." 2s. 6d.
"The Cottage Hospital Case Book or Register of Patients." 10s. and 12s. 6d.
"The Furnishing and Appliances of a Cottage Hospital." 6d.
All these are published by the Scientific Press, Ltd., and may be obtained through any bookseller, or direct from the publishers, 28 and 29 Southampton Street, Strand, London, W.C.

